# Editorial: Nutritional Buffering Strategies to Improve Exercise Capacity and Performance

**DOI:** 10.3389/fnut.2021.669102

**Published:** 2021-03-25

**Authors:** Bryan Saunders, Lars R. McNaughton, Jason Siegler

**Affiliations:** ^1^Applied Physiology and Nutrition Research Group, Rheumatology Division, Faculdade de Medicina FMUSP, School of Physical Education and Sport, University of São Paulo, São Paulo, Brazil; ^2^Institute of Orthopaedics and Traumatology, Faculty of Medicine FMUSP, University of São Paulo, São Paulo, Brazil; ^3^Sports Nutrition and Performance Group, Department of Sport and Physical Activity, Edge Hill University, Ormskirk, United Kingdom; ^4^College of Health Solutions, Arizona State University, Tempe, AZ, United States

**Keywords:** nutritional supplements, beta-alanine, sodium bicarbonate, ergogenic aid, carnosine, alkalosis

Research has demonstrated that a decline in muscle pH can inhibit contractile function and is a significant contributor within the fatigue paradigm associated with prolonged, high intensity exercise performance. A large body of work has demonstrated the efficacy of nutritional interventions, such as beta-alanine and sodium bicarbonate supplementation, that appear to support endogenous buffering mechanisms tasked with maintaining systemic pH during high rates of anaerobic metabolism, such as prolonged high-intensity exercise (1, 2). Although exogenous buffering supplementation has been studied for close to 100 years (3), numerous questions remain (both applied and mechanistic), which ultimately provided the impetus for this research topic. Collectively, Nutritional Buffering Strategies to Improve Exercise Capacity and Performance provided a consolidated platform for researchers to come together and disseminate novel data that are redefining our understanding and implementation of these nutritional strategies. This brief editorial summarizes and highlights some of these novel findings, beginning with the intracellular buffer beta-alanine.

Rezende et al. performed a systematic review and meta-analysis on studies that measured the muscle carnosine response to beta-alanine supplementation. The analytical model showed that muscle carnosine is relatively stable in skeletal muscle in the absence of supplementation. Human skeletal muscle has a large capacity for muscle carnosine accumulation and effectually all individuals respond to beta-alanine supplementation (albeit with large variability in the magnitude of the response). As further discussed by Perim et al. in their narrative review, commonly used beta-alanine supplementation protocols do not come close to saturating muscle carnosine content, providing scope to optimize supplementation strategies to increase muscle carnosine content. Therein the authors discuss modifiable factors that may better optimize the muscle carnosine response to beta-alanine supplementation including dose and duration of supplementation, supplement formulation, diet, exercise, and co-supplementation with other substances. The dose and duration of supplementation appear to be the main modifiable factors that have a substantial influence on the muscle carnosine response to supplementation, and the authors highlight appropriate recommendations to guide future research.

The importance of supplementation duration is consistent in both reviews (2, 3), as further highlighted by Ribeiro et al. This contribution demonstrated that 3 weeks of beta-alanine supplementation throughout an intense training period before an international competition did not attenuate the negative effect that training had on high-intensity intermittent exercise capacity in female footballers. Although it is unclear why exercise capacity was reduced following training, the decline in both aerobic (YoYo Intermittent Recovery Test) and anaerobic (Running Anaerobic Sprint Test & 20-m maximal sprint) capacity suggest that international teams may inadvertently overload their players during these short periods. Whether beta-alanine supplementation may attenuate this decline in capacity is still unknown given the short-duration of the supplementation period likely being sub-optimal. Future research designs might consider starting supplementation earlier to ensure sufficient increases in muscle carnosine prior to the training period.

In the field of extracellular buffering, three original studies investigated the effects of novel sodium bicarbonate supplementation strategies on various aspects of exercise performance. Boegman et al. recruited an impressive 23 world-class rowers to determine whether ingestion timing would impact a 2,000-m rowing time-trial performance. The authors demonstrated that when the time-trial commenced at a time corresponding to an individual rower's peak change in blood buffering capacity after ingesting 0.3 g·kg^−1^BM of sodium bicarbonate, their performance was improved when compared to a standardized ingestion timing of 60-min prior to exercise. Although the performance gains were small (less than a 2 s improvement), this finding may be noteworthy given the caliber of athletes included in this study cohort. Another interesting study showed that both a 0.2 and 0.3 g·kg^−1^BM dose of sodium bicarbonate may improve outcomes associated with high-intensity interval training (HIIT) even after a standardized 60-min ingestion period Gurton et al. Although both doses resulted in mild-to-moderate gastrointestinal symptoms, the ability to sustain maximal aerobic power was prolonged in a dose-response manner (~14% for 0.2 g·kg^−1^BM & ~26% for 0.2 g·kg^−1^BM) when compared to placebo. Finally, another study demonstrated the possibility that incremental doses of sodium (10, 20, 50 mmol·L^−1^) by itself may be ergogenic. These authors observed dose-response improvements in groundstroke performance in nationally-ranked British tennis players Munson et al. Performance improvements were associated with a reduction in ratings of perceived exertion, perception of thirst and gastrointestinal discomfort.

Similar to Gurton's HIIT application Gurton et al., one study also investigated the effects of sodium bicarbonate as a strategy to accelerate recovery between high-intensity exercise bouts Gough et al. Ten minutes following a boxing-specific high-intensity interval running protocol followed by a high-intensity run to exhaustion, seven elite male professional boxers ingested 0.3 g·kg^−1^BM of sodium bicarbonate. Participants then completed a boxing-specific punch combination protocol followed by another high-intensity run to exhaustion. Time-to-exhaustion in the second exhaustive exercise bout was greater with sodium bicarbonate compared to placebo (an increase of 164 ± 90 s vs. an increase of 73 ± 78 s) and was accompanied by greater recovery of acid-base balance, including blood pH and bicarbonate. These data suggest that sodium bicarbonate may also be an effective recovery tool for athletes engaged in repeated-bout activities such as boxing and combat sports.

The final study on extracellular buffers showed that the size of the capsules in which sodium bicarbonate is ingested can alter the pharmacokinetic response of blood pH and bicarbonate following supplementation Middlebrook et al. The importance of capsule size and other issues related to pharmacokinetics may have real practical relevance considering the importance of ingestion timing, as highlighted by Boegman et al. Specifically, small capsule sizes led to quicker increases and time-to-peak values of blood bicarbonate, when compared to medium and large capsules. In practice, individuals aiming to increase buffering capacity quickly might want to consider supplementing sodium bicarbonate in small capsules.

In conclusion, this research topic has resulted in a novel collection of articles that have furthered our knowledge in the area of personalized sport and exercise nutrition. We hope that the research included in this topic will act as a potent stimulus for further research in this exciting area and welcome any future topics that may build upon the evidence presented in these papers.

## Author Contributions

All authors contributed equally and approved the final version.

## Conflict of Interest

The authors declare that the research was conducted in the absence of any commercial or financial relationships that could be construed as a potential conflict of interest.
